# Restrictive and liberal red cell transfusion strategies in adult patients: reconciling clinical data with best practice

**DOI:** 10.1186/s13054-015-0912-y

**Published:** 2015-05-05

**Authors:** Marek A Mirski, Steven M Frank, Daryl J Kor, Jean-Louis Vincent, David R Holmes

**Affiliations:** Johns Hopkins Medical Institutions, 1800 Orleans Street, Phipps 455b, Baltimore, MD 21287 USA; Mayo Clinic College of Medicine, 200 First Street SW, Rochester, MN 55905 USA; Erasme Hospital, Free University of Brussels, Route de Lennik 808, 1070 Bruxelles, Belgium

## Abstract

Red blood cell (RBC) transfusion guidelines correctly promote a general restrictive transfusion approach for anemic hospitalized patients. Such recommendations have been derived from evaluation of specific patient populations, and it is important to recognize that engaging a strict guideline approach has the potential to incur harm if the clinician fails to provide a comprehensive review of the patient’s physiological status in determining the benefit and risks of transfusion. We reviewed the data in support of a restrictive or a more liberal RBC transfusion practice, and examined the quality of the datasets and manner of their interpretation to provide better context by which a physician can make a sound decision regarding RBC transfusion therapy. Reviewed studies included PubMed-cited (1974 to 2013) prospective randomized clinical trials, prospective subset analyses of randomized studies, nonrandomized controlled trials, observational case series, consecutive and nonconsecutive case series, and review articles. Prospective randomized clinical trials were acknowledged and emphasized as the best-quality evidence. The results of the analysis support that restrictive RBC transfusion practices appear safe in the hospitalized populations studied, although patients with acute coronary syndromes, traumatic brain injury and patients at risk for brain or spinal cord ischemia were not well represented in the reviewed studies. The lack of quality data regarding the purported adverse effects of RBC transfusion at best suggests that restrictive strategies are no worse than liberal strategies under the studied protocol conditions, and RBC transfusion therapy in the majority of instances represents a marker for greater severity of illness. The conclusion is that in the majority of clinical settings a restrictive RBC transfusion strategy is cost-effective, reduces the risk of adverse events specific to transfusion, and introduces no harm. In anemic patients with ongoing hemorrhage, with risk of significant bleeding, or with concurrent ischemic brain, spinal cord, or myocardium, the optimal hemoglobin transfusion trigger remains unknown. Broad-based adherence to guideline approaches of therapy must respect the individual patient condition as interpreted by comprehensive clinical review.

## Introduction

Within the ICU and other in-patient care settings, there is little debate amongst practicing physicians that promoting a restrictive transfusion strategy is reasonable in routine, stable hospitalized patients. Physicians would in the majority also agree that red blood cell (RBC) transfusion remains a life-saving intervention in those with severe bleeding and manifesting physiological indices of hypoperfusion or shock. In the center of this clinical spectrum, healthcare professionals must incorporate intelligent analysis of available clinical information regarding transfusion benefits and its risks into their care plan.

The current guideline for RBC transfusion derives from recommendations of the AABB (formerly the American Association of Blood Banks) published in 2012 [1]. This restrictive transfusion guideline states that the AABB strongly recommends adhering to a restrictive transfusion strategy hemoglobin (Hb) goal of 7 to 8 g/dl in hospitalized, stable patients. In patients with pre-existing cardiovascular disease, the recommendation is weak that transfusion should be considered for patients with symptoms or an Hb level ≤8 g/dl. The AABB does not make recommendations for or against a liberal or restrictive transfusion threshold for hospitalized, hemodynamically stable patients with an acute coronary syndrome, and weakly recommends that transfusion decisions be influenced by symptoms as well as Hb concentration.

While clinical guidelines serve an important function, two common adverse consequences of such recommendations are that they are often misapplied to patient populations outside of those intended, and secondly that there is misinterpretation of the policy itself. Applying a perfunctory review of the RBC restrictive transfusion guideline, it is easy to presume the following: that restrictive transfusion practice is routinely applicable to all care environments where blood product transfusion may be contemplated – ICU as well as perioperative and periprocedural locations; that restrictive RBC transfusion practice has been demonstrated to be beneficial to patient outcome as compared with more liberal strategies; and hence the corollary, that liberal transfusion practice is in itself harmful and thus one should refrain from transfusion when there is any doubt of its benefits. These presumptions can be viewed as logical and actionable inferences, except when careful review of the available RBC transfusion research data dictates otherwise. For patient safety and benefit, misconceptions must be avoided. Physicians need to be encouraged to treat the patient and the clinical circumstance, not simply the Hb, thereby limiting or applying RBC transfusion therapy based on the patient’s physiological status or situational risk of incurring severe hemorrhage and organ ischemia.

While the RBC transfusion recommendations are fundamentally well founded, there remain limitations in our understanding of the indications, risks, and benefits of such transfusion. These knowledge gaps are particularly prevalent in the gray zones of RBC transfusion practice, such as in the setting of active bleeding, the potential for end-organ ischemia (for example, myocardial ischemia, septic shock), and neurologic illness and injury. Such gaps raise serious concern when adherence to the restrictive transfusion guidelines is broadly promulgated in the current clinical literature, emphasizing a broad endorsement of the guideline. To suggest that such a view is unfounded, it may be noted that broad-based measures have been recently applied in assessing compliance with the aforementioned restrictive guidelines. While the decision to transfuse RBC should be based upon a comprehensive, patient-specific clinical assessment, deviation from the target of such published compliance measures has been specifically criticized as failure of their adherence [2].

To provide the physician with a RBC transfusion compass, we reviewed the clinical research data in support of a restrictive or a more liberal RBC transfusion practice, and examined the quality and content of the datasets. Reviewed studies included PubMed-cited (1974 to 2013) prospective randomized clinical trials (RCTs), prospective subset analyses of randomized studies, nonrandomized controlled trials, observational case series, consecutive and nonconsecutive case series, and review articles. Prospective RCTs were acknowledged and emphasized as the best-quality evidence. We have organized the review to provide a synopsis of RBC transfusion trial data, risks of anemia and transfusion, concerns in the bleeding versus nonbleeding patient, and specific issues regarding RBC transfusion that arise in one common clinical scenario – interventional cardiology.

## Red blood cell transfusion strategies: premise and data

The series of recommendations by the AABB regarding the RBC transfusion threshold have their principal origin in the large published clinical trials (seven trials each being Level 1, >400 patients) that have demonstrated the relative safety of a restrictive transfusion strategy (Hb transfusion threshold <7 g/dl) in specific clinical settings in adult cohorts [3-9]. The large RCTs have evaluated Hb-based RBC transfusion thresholds in several study populations, including patients in mixed medical/surgical ICUs [3,5], patients undergoing cardiac and orthopedic surgery [5,7], patients with sepsis [8], and patients experiencing upper gastrointestinal bleeding [9] (Table 1). These hypothesis-driven trials have proposed that restrictive RBC transfusion strategies are as safe as, or perhaps more safe than, liberal transfusion practices. To evaluate the primary hypotheses proposed in these trials, explicit potential adverse effects of RBC transfusion were also evaluated, aiming to demonstrate that guideline compliance would lead to a reduction in a number of transfusion-related adverse effects, of which the most commonly cited include hospital-acquired infection, transfusion reaction (severe and mild incompatibility), transfusion-associated circulatory overload (TACO), transfusion-related acute lung injury, and immunomodulation, all of which could lead to an increase in patient morbidity and mortality [1].

**Table 1:** **Large prospective randomized clinical trials on transfusion triggers**

**Clinical trial**	**Patient population**	**Restrictive strategy (Hb trigger – target) (g/dl)**	**Liberal strategy (Hb trigger – target) (g/dl)**	**Reduction in blood utilization**	**Primary outcome**			
					**Event**	**Restrictive strategy incidence (%)**	**Liberal strategy incidence (%)**	***P*** **value**
Hébert and colleagues [3] (*n* = 838)	Critically ill (adults)	7 to 8.5	10 to 10.7	54% less RBC units transfused	30-day mortality	18.7	23.3%	0.11
Hajjar and colleagues [5] (*n* = 502)	Cardiac surgery (adults)	8 to 9.1	10 to 10.5	58% less RBC units transfused	Composite endpoint	11	105	0.85
					• 30-day mortality	6	6	0.93
					• Cardiogenic shock	9	1	0.42
					• ARDS	2	5	0.99
					• Acute renal injury requiring dialysis	4		0.99
Carson and colleagues [7] (*n* = 2,016)	Femur fracture (older adults)	8.0 to 9.5	10.0 to 11.0	65% less RBC units transfused	Composite endpoint	34.7	35.2	NS
					• 60-day mortality	28.1	27.6	NS
					• 60-day inability to walk	6.6	7.6	NS
Villanueva and colleagues [9] (*n* = 921)	Gastrointestinal bleeding (adults)	7 to 9.2	9 to 10.1	59% less RBC units transfused	45-day all-cause mortality	5	9	0.02
Holst and colleagues (*n* = 998) [8]	Sepsis in the ICU (adults)	<7.0	7.0 to 9.0	50% less RBC units transfused	90-day all-cause mortality	43	45	0.44

ARDS, acute respiratory distress syndrome; Hb, hemoglobin; NS, nonsignificant; RBC, red blood cell.

Having the hypothesis that a restrictive transfusion regimen would offer medical benefit, especially to that population of sicker, older patients, Hébert and co-investigators of the landmark 1999 Transfusion Requirements in Critical Care (TRICC) trial acknowledged at the trial’s end that there was no difference between liberal and restrictive cohorts in the only primary outcome measure of all-cause 30-day mortality [3]. A secondary outcome measure, in-hospital mortality, was lower in the restrictive group (*P* = 0.05) but in the context of no mortality benefit during the ICU stay or at 60 days. Three of four mortality risk assessments were thus negative between cohorts, including the shortest and longest time spans evaluating patient survival. Interestingly, and counter to their original hypothesis, subgroup analysis revealed that restrictive RBC transfusion was associated with reduced mortality in younger not older study participants (age <55) and in those with less severe acute illness (Acute Physiology and Chronic Health Evaluation II scores <20) [3]. If this observation was true, then the opposite comparison must also be valid; namely that liberal transfusion strategy reduced mortality in the older and very ill patient (data were not specifically reported in the study), because the overall mortality rates between transfusion strategies were not different. The finding was probably a statistical quirk, and subsequent trials evaluating leukocyte-reduced RBCs have generally not confirmed a mortality benefit with restrictive RBC transfusion strategies (Table 1).

When taken in aggregate, however, the available large clinical trial data clearly support non-inferiority of restrictive RBC transfusion practices in the acute setting in stable hospitalized patients. Together with additional observational and correlative studies that link potential harm and worse clinical outcome to RBC transfusion [10-23], many published reviews and international medical societies have therefore endorsed a Hb transfusion trigger of approximately 7 g/dl as a practice guideline for most hospitalized and stable critically ill patients [24-27]. Such recommendations no doubt reflect the desire for patient wellbeing together with optimal stewardship of a valuable resource. There is little debate regarding the important economic and societal considerations of minimizing unwarranted transfusions; regional or national blood shortages in the USA are reported with regularity, and the $200 to 250 per RBC unit hospital acquisition fee is but a fraction of the activity-based cost of transferring a blood unit from the donor to the recipient, estimated to be $500 to 1,200 per RBC unit [28].

Nevertheless, it is important to note that these very same studies have largely failed to show actual physiological benefit over more liberal transfusion practices. Furthermore, the correlation between transfusion and a variety of adverse effects emanate almost exclusively from observational datasets, not Level 1 studies, which have linked RBC transfusion with the risk for increased mortality, suppressive immunomodulation, nosocomial infections, ischemic complications, and acute kidney injury (Table 2) [10-23]. Although the aggregate of the observational clinical data does raise concern regarding these potential associations, the pervasive risk for uncontrolled or unmeasured bias and confounding errors of analysis preclude cause-and-effect determinations. Such research by association must be interpreted with great caution. In the vast majority of cases, transfusion as a specific intervention merely serves as a marker for increased disease complexity. As suggested by Doyle and colleagues, who reviewed the potential link between transfusion and mortality from percutaneous coronary intervention (PCI), and who recognized the strong correlation between co-morbidities and bleeding; ‘no amount of statistical adjustment can provide certainty of a cause-and-effect relationship when there is this degree of correlation’ [29]. If we were to summarize the observational outcome studies in aggregate, the conclusions reached would be: anemia, bleeding, and transfusion are all detrimental. To better guide the clinician in circumstances of anemia, active bleeding, or in a patient at risk for hemorrhage, accurate interpretation of the current data pool is warranted.

**Table 2:** **Strength of association between red cell transfusion and purported clinical adverse effect**

	**Highest-level clinical studies**			
**Adverse effect**	**Showing correlating effect**	**References**	**Showing no correlation**	**References**
Multisystem organ dysfunction	Observational studies	[11,12]	Level 1 RCT	[3,8]
Nosocomial infection	Observational and retrospective studies	[11,13]	Level 1 RCT (2), RCT (1), RCT meta-analysis	[1,4,5,8,9]
Allergic or immunomodulation, tumor promotion	Observational and retrospective studies	[14-16]	Level 1 RCT (2)	[8,9]
Pulmonary edema	Level 1 RCT	[3,9]	Level 1 RCT (3), RCT meta-analysis	[1,5,7-9]
Pulmonary (non-edema) including ARDS	Observational studies	[11,17]	Level 1 RCT (3), RCT (1)	[4,5,7-9]
Acute kidney injury	Observational and retrospective studies	[18,19]	Level 1 RCT (2), RCT (1)	[4,5,8,9]
Myocardial ischemia	Prospective cohort	[20,21]	Level 1 RCT (4), RCT (1)	[3-5,7-9]
Cerebral ischemia	Observational and retrospective studies	[10,20,22]	Level 1 RCT (3), RCT (1), RCT meta-analysis	[1,4,5,7-9]
Shock	Observational study	[11]	Level 1 RCT	[3,5,8]
Cardiac arrest	Prospective cohorts	[20,21]	Level 1 RCT	[3,5,7,8]
Bleeding/coagulopathy	Observational study	[11]	Level 1 RCT	[5,8]

Highest-level study reflects the rank order of scientific merit typically afforded to studies based on trial design. Highest to lowest: prospective RCTs, prospective subset analyses of randomized studies, nonrandomized controlled trials, observational case series (including prospective and retrospective cohort analysis), and consecutive and nonconsecutive case series. ARDS, acute respiratory distress syndrome; RCT, randomized clinical trial.

Thankfully at this time, owing an enormous debt to blood-banking quality assurance, transfusion errors in storage and compatibility as well as the risk of viral infection are now extraordinarily low, with each having a risk of <1/100,000 to 2,000,000 [1]. Also uncommon, the incidence rate of transfusion-related acute lung injury is approximately 0.81/10,000 transfused units in well-defined cases, or roughly 0.01% of patients transfused [30-32]. This risk is similar to the expected frequency of being killed in a motor vehicle accident or from a fall [1], but nonetheless remains the leading cause of transfusion-related death reported to the US Food and Drug Administration. In contrast, TACO appears with more regularity, with an estimated incidence rate ranging from 1 to 8% with a mortality hazard ratio of 3.20 (95% confidence interval (CI), 1.23 to 8.10) [1]. The precipitation of TACO, however, is more a consequence of the manner in which the transfusion is delivered rather than a direct toxicity of blood component therapy, consistent with it being known to occur at a higher rate among those who are transfused with more units of RBCs.

Interestingly, inspection of the RCTs themselves reveals that, with the exception of TACO, liberal RBC transfusion practices have not been readily associated with increased rates of transfusion-related complications [3-9]. Specifically, with the exception of the recently reported trial in upper gastrointestinal bleeding where brisk hemorrhage impacted upon survival [9], there has been no overall greater mortality risk or significant increases in the rates of multisystem organ dysfunction, nosocomial infection, acute kidney injury, coagulopathy, immune disorder, acute respiratory distress syndrome or pneumonia, or ischemic complications such as myocardial infarction or stroke in review of primary outcome data [3-9] (Table 2). One must ask: why do the results of these clinical trials not support the substantial body of observational data? There are two principal explanations. First, most serious transfusion-related complications are rare events; hence the number of patients required to detect statistically significant differences in rates of complications between restrictive and liberally transfused cohorts would preclude study feasibility. It is fair to say that the RCTs in general were not powered to recognize uncommon adverse events. A second and more profound explanation relates to the inescapable limitation of all observation studies; namely the findings simply identify associations, not cause-and-effect relationships. In effect, RBC transfusion simply becomes a marker for greater severity of illness. Indeed, the conclusion as per the primary outcome metrics from such studies as the TRICC trial, and the follow-up subanalysis examinations in trauma patients [33] and cardiac patients [34], as well as in the domains of ICU, orthopedics [7], and cardiac surgery [5], is that restrictive strategies in nonhemorrhagic patients are no worse than liberal strategies under the limited protocol study conditions.

If RBC transfusions do precipitate a substantial risk of adverse effects, the incidence of such events should be highest in patients receiving the greatest amounts of allogeneic blood. Yet data from large (≥1,000 patients) ICU studies have generally failed to demonstrate such a relationship [3,8,35,36]. In addition, in both military and domestic studies of patients during massive active hemorrhage or with the risk of hemorrhage, there are data to support that aggressive resuscitation with RBCs and other blood component therapy does not correlate with increased risk, and may lead to a decrease in both morbidity and mortality as compared with lower Hb-targeted transfusion thresholds. During Operation Iraqi Freedom and Enduring Freedom, the number of transfused RBCs, plasma, and platelets in a registry of >3,000 patients actually increased per trauma incident over time, while crystalloid administration decreased [37]. Adhering to such a massive transfusion protocol, mortality was lower (*P* <0.001) despite higher injury scores during latter years. These data have been replicated in a 600-patient cohort in domestic trauma [38] as well as in other large clinical studies [39,40]. However, there is also a clear association between high-volume blood component therapy and adverse events linked to each of the three components [41-43]. Hence, the optimal ratio for such a massive transfusion protocol has yet to be determined. Most probably, such a transfusion response will need to be individualized to each clinical event to minimize transfusion complications.

## Anemia and red blood cell efficacy

Anemia has long been associated with adverse patient outcomes. With respect to correlative data, anemia in older people has been well documented as being an independent risk factor for increased mortality, functional dependence, impaired cognition, re-admission to hospital, and falls [44-46]. The challenge remains to elucidate at what risk–benefit point the treatment of anemia with transfusion will be associated with net improvement in functional capacity and outcome. The large prospective restrictive versus liberal transfusion studies evaluated select populations over a short period of time, typically ICU or hospital lengths of stay, with no assessment of ultimate functional outcome or rate of recovery.

In one well-conducted prospective study with 1,156 patients >65 years old who were evaluated in six basic and eight instrumental activities of daily living, anemia as defined by Hb <12.0 g/dl incurred poorer performance and strength [47]. Similarly, Foss and colleagues prospectively demonstrated in a population of 487 hip fracture patients that a more severe degree of anemia (<10 g/dl) was associated with a linear Hb concentration dependency with respect to independent ambulation on each of the 3 days postoperatively (16% first day, 25% second day, 40% third day with no anemia versus 5% first day, 18% second day, 26% third day with anemia, *P* < 0.001), with a significantly increased 30-day mortality of 12.6% versus 6.3% (*P* < 0.05), and with an increased length of hospital stay (13 days (9–23) versus 8 days (6–18) (*P* < 0.001)) [48]. In opposition to these data, the more recent, prospective randomized Functional Outcomes in Cardiovascular Patients Undergoing Surgical Hip Fracture Repair (FOCUS) trial demonstrated that a liberal transfusion strategy offered no overall benefit in terms of reducing mortality or the ability to ambulate without assistance 60 days following surgery [7]. However, strength, endurance, and other metrics of robust, independent function were not measured. Additionally, the primary functional outcome of ambulation was defined as the ability to walk a distance of 10 feet, which is not a robust measure of either stability or endurance. For such assessment, either the 10-meter walk or the 6-minute walk test is incorporated for such purpose as standard evaluation metrics in the practice of rehabilitative medicine [49,50].

## The perioperative or bleeding patient

The FOCUS trial by Carson and colleagues was conducted on patients undergoing hip replacement surgery, in which patients were randomized to a restrictive (Hb threshold 8 g/dl) or liberal (Hb threshold 10 g/dl) transfusion during and following surgery [7]. As expected, more RBC units were transfused in the liberal group; it must be presumed for resuscitation purposes that greater amounts of other intravenous crystalloids or colloids were required and used in the restrictive strategy cohort, although these data were not provided. Acute RBC transfusions, however, were required in 14 patients within the restrictive strategy cohort for rapid bleeding (versus five patients in the liberal cohort, *P* < 0.05). In addition, transfusions were necessary to manage acute congestive heart failure in 10-fold the patient number in the restrictively treated group (*P* < 0.001), and transfusions were required for acute hypotension or tachycardia in greater than 12% (versus 4.3%) of the restrictive RBC-managed patients (*P* < 0.001) [7]. To summarize, such permissive anemia management during the perioperative period led to more acute fluid interventions and RBC transfusion, and more diuresis and intensive respiratory treatments were required to avoid any further deleterious adverse events resulting specifically from a low Hb-fluid resuscitated condition. The lesson is not that a restrictive RBC transfusion strategy has no place when considering surgery. Rather, in a protocol trial, considerations were not made (or could not be made in this case) to better discriminate which patients were at risk for Hb reduction to levels below normal physiological limits. Indeed, a serum Hb of 8 g/dl equates to a >40% decrement from a routine preoperative concentration of 13 to 14 g/dl. To better ascertain the safety of such anemia, each patient deserves a comprehensive physiological assessment to chart their clinical management strategy, and not simply processed along a broad guideline strategy, as was in fact required by the tenets of this randomized trial.

Similarly, the study by Villanueva and colleagues was the first, and remains the only, major prospective trial comparing restrictive versus liberal RBC transfusion strategies in patients with ongoing nonsurgical hemorrhage (gastrointestinal bleeding) [9]. With regards to their primary outcome measure of mortality at 45 days, the authors published that the death rate was significantly lower in the restrictive strategy cohort (target Hb 7 to 9 g/dl) than it was in the liberal strategy group (9 to 11 g/dl): 5.2% (23 of 444 patients) as compared with 9.2% (41 of 445 patients), respectively (*P* = 0.02).

The implications of this study are profound, as the authors extended the recommendations of the restrictive RBC transfusion guidelines to patients with active hemorrhage. Hence it is important to review the raw data and the conclusions in more detail. In the study, patient death due to initial unsuccessful controlled bleeding was not equally distributed between the restrictive and liberal transfusion strategy groups: three patients (0.7%) in the restrictive strategy group versus 14 patients (3.1%) in the liberal strategy group (*P* = 0.01). Simply by equalizing the patient number in this category in which death and transfusion were entirely unrelated would have rendered a statistically insignificant result to the primary outcome measure of overall mortality. Of note, neither platelet or plasma transfusions coincided with more RBCs administered, nor was the total volume of intravenous fluids less per patient in the liberal transfusion strategy cohort, so a desire to attain a higher Hb goal did not reduce the clinicians’ use in this study of crystalloids and colloids. This result probably explains why TACO was the most significant adverse event observed between the two groups. As a consequence of reduced transfusion rates, more patients in the restrictive transfusion group (45%) incurred Hb levels <7 g/dl at some point during their hospitalization compared with the liberally transfused group (18%) (*P* < 0.001), which is of potential concern in patients with symptomatic cardiovascular or neurological disease.

In addition to hemorrhagic shock, the risk of withholding blood replacement therapy during active hemorrhage remains a cogent issue. The measured Hb at the time of hemorrhage fails to accurately reflect true red cell mass or oxygen-carrying capacity. Rather, it is not until the lost red cell mass is replaced with non-RBC replacement solutions (for example, crystalloids and/or colloids) that the decrease in Hb concentration can be accurately measured. Such volume replacement therapy is imperative in a patient with intravascular depletion and carries its own adverse risks. Some of the concerns with regards to the transfusion of RBCs are shared by crystalloid volume replacement – volume overload, electrolyte derangement, hypothermia, and coagulopathy. Replacing shed blood with intravenous fluids/colloids therefore carries increased morbidity and can be life-threatening [51-53], and is not a panacea to withholding blood product transfusion. The bridge here, between clear benefit to transfusion versus that of a restrictive algorithm, is in knowing when a patient is at risk for a serious hemorrhage and a consequent severe anemic state (Hb <6 to 7 g/dl). In circumstances of high risk, or when volume resuscitation is required, the hazard of developing progressive anemia, end-organ ischemia, or fluid overload should be considered, and adherence to restrictive or Hb-based transfusion strategies is not only suboptimal but outside the premise of the guideline.

## Clinical navigation

The documented equivalence between liberal and restrictive RBC transfusion practices certainly supports a restrictive approach in the majority of hemodynamically stable, nonbleeding patients. The guidelines proffer a strong endorsement to limit RBC transfusion, yet offer weak emphasis on careful patient discrimination and the risks of not offering transfusion – which include more crystalloid and risk of pulmonary congestion and cardiac strain. It must be reinforced that in the larger, prospective restrictive versus liberal transfusion investigations, pretrial patient selection was not heterogeneous because only a limited number of patients with acute coronary syndromes, traumatic brain injury, or neurosurgery patients at risk for brain or spinal cord ischemia were included in these trials.

In both cardiac surgery trials [4,5], as well as the small (*n* = 45) myocardial infarction study by Cooper and colleagues [6], patients with myocardial disease were evaluated with good overall tolerance to a restrictive transfusion algorithm. Transfusion effects during symptomatic disease were not examined, however, and a subgroup analysis of the TRICC trial injected some caution into withholding RBC transfusion in a subset population with severe coronary disease [34]. Furthermore, in a recent pilot study by Carson and colleagues, patients undergoing cardiac catheterization for acute coronary syndrome or stable angina had a trend towards a reduction in both mortality and cardiac events after randomization to a liberal (10 g/dl) as compared with a restrictive (8 g/dl) transfusion trigger [54]. The authors concluded that a formal, adequately powered trial was necessary to confirm the results. In the neuroscience realm, clinicians are not surprisingly concerned regarding the effect of anemia on the low neurological threshold to hypoxia/ischemia, as brain and spinal cord tissues each offer little anaerobic reserve. Studies in patients with traumatic brain injury and subarachnoid hemorrhage generally support that reduced oxygen delivery as manifested by a 30 to 40% reduction in Hb concentration (Hb 8 to 9 g/dl) may be hazardous [55,56]. Similarly, the potential benefits of higher Hb levels within the domains of geriatric medicine and medical–surgical physical rehabilitation, where assessment of longer term functional outcomes are important, are often not addressed when discussing restrictive transfusion [44-48].

The physiological utility of oxygen delivery via RBC transfusion cannot be ignored and remains a salient issue. At rest, myocardial energy demands are high, with coronary extraction of oxygen approaching 90%, with virtually no measurable increase during conditions of ischemia [57]. During anemic states (Hb <10 g/dl), systemic oxygen delivery is maintained via initial increases in stroke volume and then heart rate, which leads to early subendocardial ischemia when coronary sinus oxygen supply is diminished. Such conditions of energy imbalance are further increased in patients with atherosclerotic or valvular disease [57].

An important RBC storage lesion often discussed is depletion of 2,3-diphosphoglycerate, whose level is inversely proportional to the affinity of Hb for oxygen [58]. Despite an increase in Hb following a transfusion, left-shifting the Hb disassociation curve may theoretically not improve tissue delivery of oxygen. However, there are ample clinical data during correction of anemia in both stable and critically ill patients that support an acute increase in oxygen delivery and functional organ tissue benefit following RBC transfusion [59-61]. This effect also appears independent of the storage duration of the transfused red cells [60-62]. It has been hypothesized that oxygen delivery is reduced less than predicted because the oxygen affinity of Hb is affected also by temperature, pH, and base excess, and it is the local *in vivo* environment with respect to these variables that may reduce the impact of the decreased 2,3-diphosphoglycerate [60].

In patients with cardiac disease and high risk for ischemia, anemia may have substantial consequences. Often cited is the study by Rao and colleagues, which demonstrated that, on reviewing data for the more than 24,000 patients enrolled in the GUSTO IIb, PURSUIT, and PARAGON B trials, transfusion – as the fixed variable of study – was associated with an increased hazard for 30-day death (adjusted hazard ratio, 3.94; 95% CI, 3.26 to 4.75) and 30-day death/myocardial infarction (adjusted hazard ratio, 2.92; 95% CI, 2.55 to 3.35) [20]. In these trials of active intervention, however, transfusion was no doubt a marker for hemorrhage and a deteriorating patient, because no threshold trigger was otherwise invoked. In a more recent study involving review of adverse cardiovascular events through 30 days in 39,922 patients enrolled in clinical trials of acute coronary syndromes, when Hb was the fixed variable – not transfusion – cardiovascular mortality increased as Hb levels fell below 14 g/dl, with an adjusted odds ratio of 1.21 (95% CI 1.12 to 1.30, *P* < 0.001) for each 1 g/dl decrement in Hb [63]. To further this point, in the recent Integrilin and Enoxaparin Randomized Assessment of Acute Coronary Syndrome Treatment trial, the corresponding rates of recurrent ischemia were 39.1%, 22.0%, 15.6%, and 11.9% in patient cohorts that had base Hb levels of <12.0 g/dl, 12.0 to 13.9 g/dl, 14.0 to 15.9 g/dl, and >16.0 g/dl (*P* = 0.004) [64].

In a study of 3,600 patients published last year examining the specific relationship of Hb level and postoperative myocardial ischemia, the results showed that postoperative Hb levels within the intermediate tertile (10.3 to 11.6 g/dl) (odds ratio, 5.8; 95% CI, 1.6 to 20.9) and the lowest tertile (7.9 to 10.2 g/dl) (odds ratio, 12.9; 95% CI, 3.0 to 55.5) carried the highest odds-ratio risk for coronary ischemia, greater than even a prior history of ischemic heart disease (odds ratio, 4.2; 95% CI, 1.0 to 17.8) [65].

## Clinical example – interventional cardiology

Perhaps in no other recent area of clinical practice has there been as much interest in the role of anemia and RBC transfusion as in patients with cardiovascular disease undergoing PCI – either for stable coronary artery disease or an acute ischemic syndrome. This domain has received the highest attention with regards to bleeding and transfusion, with over 100 studies and reports during the past 2 years alone [66-70]. Although bleeding following PCI has been clearly associated in prospective cohorts with increased mortality [71], studies confined to the assessment of transfusion have documented such correlation using retrospective registry data [72,73]. In a study at the Mayo Clinic, 17,901 patients undergoing PCI had follow-up to 6 years, with increased mortality observed in patients who received as little as 1 or 2 units of blood as compared with patients who did not receive transfusion [74]. It is important to recognize that transfusion therapy in such studies was compared with adverse outcome simply as a bivariate risk. Physicians ordered RBCs based on their impression of the severity of need. In an uncontrolled setting, transfusion is inextricably tied to hemorrhage, and the latter is a harbinger for adverse outcome. Indeed, a summary from four major prospective randomized trials found that bleeding severe enough to reduce baseline Hb by ≥3 g/dl within 30 days following PCI was an independent predictor of 1-year mortality (incidence 15.2%), comparable with a nonfatal myocardial infarction (11.3%) [68]. Even in the absence of acute hemorrhage, the sole presence of anemia during the period of PCI represents an independent risk for poor outcome [75-79].

Another confounding variable within interventional cardiology is the use of antiplatelet agents, particularly dual antiplatelet therapy, which has been felt necessary to minimize stent thrombosis after PCI. In patients with bleeding, dual antiplatelet therapy may be discontinued early to reduce hemorrhage, thus exposing them to the threat of stent thrombosis and a consequent 40 to 50% risk of death or myocardial infarction [80]. Not surprisingly, in examination of the GRACE and PARIS registries of patients treated for acute myocardial infarction and PCI [79,80], those who bled had a higher frequency of having their antiplatelet and anticoagulant therapy discontinued, and incurred higher in-hospital mortality [81,82]. Such associations in the cardiovascular arena have led to multiple developments in an attempt to decrease both short-term and long-term mortality associated with hemorrhage, including increasing use of radial approaches for cardiac catheterization, amended transfusion thresholds, new anticoagulant regimens and vascular closure devices, and revisions to Professional Society guidelines.

Innovations in PCI may also create greater hazards with respect to hemorrhagic complications. The recent introduction of transcatheter aortic valve replacement has been approved for patients at high risk or who are deemed inoperable for conventional aortic valve replacement. At present, the use of large arterial sheaths is required, and both bleeding and vascular complications have been frequent in 7 to 10% of patients [83]. Given the frailty of many such patients, raising – not lowering – the Hb transfusion trigger may improve physiological and functional outcome. Such a hypothesis would require evaluation – a formal prospective study, not a clinical association.

## Future direction

At an academic level, a multidisciplinary think-tank from the National Heart, Lung, and Blood Institute convened and agreed to the need for three adult trials evaluating RBC transfusion trigger strategies to improve overall outcome and to validate the following hypotheses: first, higher Hb levels resulting from a liberal transfusion strategy during cardiopulmonary bypass surgery will lead to lower incidence of 30-day all-cause mortality, recurrent myocardial infarction, infection, and other complications as compared with a restrictive transfusion threshold; second, in patients with acute coronary syndrome or coronary artery disease undergoing cardiac catheterization, a liberal transfusion strategy will be associated with a lower incidence of composite outcome of all-cause mortality at 30 days, recurrent myocardial infarction, emergent percutaneous intervention (angioplasty or stent insertion), or coronary artery bypass graft surgery within 30 days of enrollment when compared with a restrictive transfusion strategy; and third, in hemodynamically stable patients in the ICU with a history of ischemic heart disease, multiple organ dysfunction scores will be improved by maintaining Hb >10 g/dl [84]. That the National Heart, Lung, and Blood Institute would propose such studies suggests that the ideal Hb transfusion trigger has yet to be determined for some common clinical conditions, and Hb levels >8 g/dl in some stable patients may be warranted.

At the level of the practicing physician, what are clinicians to do? Fundamentally, physicians should intelligently apply their skill set to their patients’ needs, incorporating all physiological data at their disposal, and incorporate – as applicable – the scientific support from Practice Guidelines. Recent survey data demonstrating the lack of a substantial reduction in ICU RBC transfusion in one state has been interpreted to suggest that restrictive RBC transfusion thresholds may not have ‘fully translated into practice in different hospital settings’ [2]. Perhaps in assessing this lack of conformity the authors of such uncontrolled, hospital-derived datasets should in part acknowledge that experienced clinical decision-making may have presided over the selection for or against transfusion. After all, the primary methodology for evaluating the restrictive transfusion hypothesis in the TRICC and FOCUS trials has been itself criticized for lack of inclusion of a usual practice arm in the dichotomized trial protocols, thus encouraging practice misalignments and hence more limited data interpretability [85]. As an optimal use of a practice guideline, trial data supportive of a restrictive transfusion strategy provide clinicians with a helpful metaphorical arm rest, to pause and carefully consider whether a transfusion is likely to be helpful, which in many cases it will not.

## Conclusion

Current evidence suggests that in many clinical settings a restrictive RBC transfusion strategy is cost-effective, reduces the risk of adverse events specific to transfusion, and introduces no harm. Similarly, implementation of restrictive, Hb-based RBC transfusion practices in bleeding patients – or those at high risk for hemorrhage – may be hazardous. In patients with ischemic brain, spinal cord, or myocardium, or in debilitated patients requiring prolonged functional recovery, the optimal Hb transfusion trigger remains unknown, but may be higher than for a severe, restrictive algorithm. Until future studies further define the role of RBC transfusion in these specific clinical scenarios, a restrictive transfusion strategy should be recommended within the well-studied patient populations and clinical conditions, and the clinicians must continue to use their experience and bedside clinical judgment to advocate the best management for their patients.

## Abbreviations

CI: confidence interval
FOCUS: Functional Outcomes in Cardiovascular Patients Undergoing Surgical Hip Fracture Repair
Hb: hemoglobin
PCI: percutaneous coronary intervention
RBC: red blood cell
RCT: randomized clinical trial
TACO: transfusion-associated circulatory overload
TRICC: Transfusion Requirements in Critical Care
